# The loss of the PDIM/PGL virulence lipids causes differential secretion of ESX-1 substrates in *Mycobacterium marinum*

**DOI:** 10.1128/msphere.00005-24

**Published:** 2024-04-25

**Authors:** Bradley S. Jones, Daniel D. Hu, Kathleen R. Nicholson, Rachel M. Cronin, Simon D. Weaver, Matthew M. Champion, Patricia A. Champion

**Affiliations:** 1 Department of Biological Sciences, University of Notre Dame, Notre Dame, Indiana, USA; 2 Eck Institute for Global Health, University of Notre Dame, Notre Dame, Indiana, USA; 3 Department of Chemistry and Biochemistry, University of Notre Dame, Notre Dame, Indiana, USA; University of Kentucky College of Medicine, Lexington, Kentucky, USA

**Keywords:** *Mycobacterium*, ESX-1, PDIM, secretion, proteomics

## Abstract

The mycobacterial cell envelope is a major virulence determinant in pathogenic mycobacteria. Specific outer lipids play roles in pathogenesis, modulating the immune system and promoting the secretion of virulence factors. ESX-1 (ESAT-6 system-1) is a conserved protein secretion system required for mycobacterial pathogenesis. Previous studies revealed that mycobacterial strains lacking the outer lipid PDIM have impaired ESX-1 function during laboratory growth and infection. The mechanisms underlying changes in ESX-1 function are unknown. We used a proteo-genetic approach to measure phthiocerol dimycocerosate (PDIM)- and phenolic glycolipid (PGL)-dependent protein secretion in *M. marinum,* a non-tubercular mycobacterial pathogen that causes tuberculosis-like disease in ectothermic animals. Importantly, *M. marinum* is a well-established model for mycobacterial pathogenesis. Our findings showed that *M. marinum* strains without PDIM and PGL showed specific, significant reductions in protein secretion compared to the WT and complemented strains. We recently established a hierarchy for the secretion of ESX-1 substrates in four (I–IV) groups. Loss of PDIM differentially impacted secretion of Group III and IV ESX-1 substrates, which are likely the effectors of pathogenesis. Our data suggest that the altered secretion of specific ESX-1 substrates is responsible for the observed ESX-1-related effects in PDIM-deficient strains.

**IMPORTANCE:**

*Mycobacterium tuberculosis,* the cause of human tuberculosis, killed an estimated 1.3 million people in 2022. Non-tubercular mycobacterial species cause acute and chronic human infections. Understanding how these bacteria cause disease is critical. Lipids in the cell envelope are essential for mycobacteria to interact with the host and promote disease. Strains lacking outer lipids are attenuated for infection, but the reasons are unclear. Our research aims to identify a mechanism for attenuation of mycobacterial strains without the PDIM and PGL outer lipids in *M. marinum*. These findings will enhance our understanding of the importance of lipids in pathogenesis and how these lipids contribute to other established virulence mechanisms.

## INTRODUCTION

Phthiocerol dimycocerosate (PDIM) and phenolic glycolipid (PGL) are outer lipid virulence factors in the mycolate outer membrane (MOM) (1–3). Although the precise role of PDIM/PGL in mycobacterial virulence remains elusive, PDIM/PGL support the cell envelope structure, serve as a permeability barrier, enhance antibiotic tolerance, spread into host membranes, and directly modulate the immune system (4–8). These lipids recruit macrophages permissive for bacterial growth and survival while promoting the evasion of bactericidal macrophages (1, 3, 6, 9–11).

PDIM and PGL are synthesized from cytoplasmic fatty acids via a series of discrete steps by the PpsA-E/FadD26, Mas/FadD28, and PapA5 enzymes (12–14). Their transport across the cytoplasmic membrane (CM) and mycobacterial arabinogalactan peptidoglycan complex (mAGP) relies on the MmpL7 and DrrABC transporters and the LppX lipoprotein (1, 3, 15–18), although the precise mechanisms remain unknown.

Mycobacteria secrete proteins across the MOM to the cell surface and into the surrounding environment, both during laboratory growth and infection (19–23). Proteins are exported across the CM by the Sec and SecA2 pathways, twin arginine transporter (TAT), and ESAT-6 (ESX) systems (24, 25). Yet the mechanisms used by proteins to cross the impermeable mAGP and the MOM (26–28) are elusive. Recent studies suggest that PE/PPE and Esx (WXG_100) proteins facilitate protein translocation across the MOM (29–31).

Early during infection, mycobacterial secreted proteins promote the interaction with host membranes and macrophage signaling pathways. The ESX-1 system secretes proteins (32) that contribute to phagosomal damage (33–35), leading to pathogen release, along with its DNA, RNA, and secreted proteins, into the macrophage cytoplasm (11, 35–37). Secreted proteins in the cytoplasm hinder phagosomal repair, affect host trafficking, and promote dissemination of infected macrophages in the lungs (35, 38–43). Mycobacterial DNA in the cytoplasm triggers the Type I IFN response (34, 36, 37, 44), promoting macrophage cell death and bacterial spread (34, 45).

In the absence of PDIM, ESX-1 function is reduced, affecting the Type I IFN response and phagosomal lysis (46, 47). It is unclear whether PDIM is required for secretion of ESX-1 protein across the MOM (46) or for ESX-1 substrate function within the phagosome (47). Our study uses a proteogenetic approach (48) to quantify how PDIM and PGL influence protein secretion from *Mycobacterium marinum*. Our studies provide an explanation for ESX-1 phenotypes in the absence of PDIM, suggesting a broader role of PDIM and PGL outer lipids in translocating mycobacterial proteins across the MOM.

## RESULTS

### Generation and characterization of PDIM-deficient *M. marinum* strains

**Fig 1:**
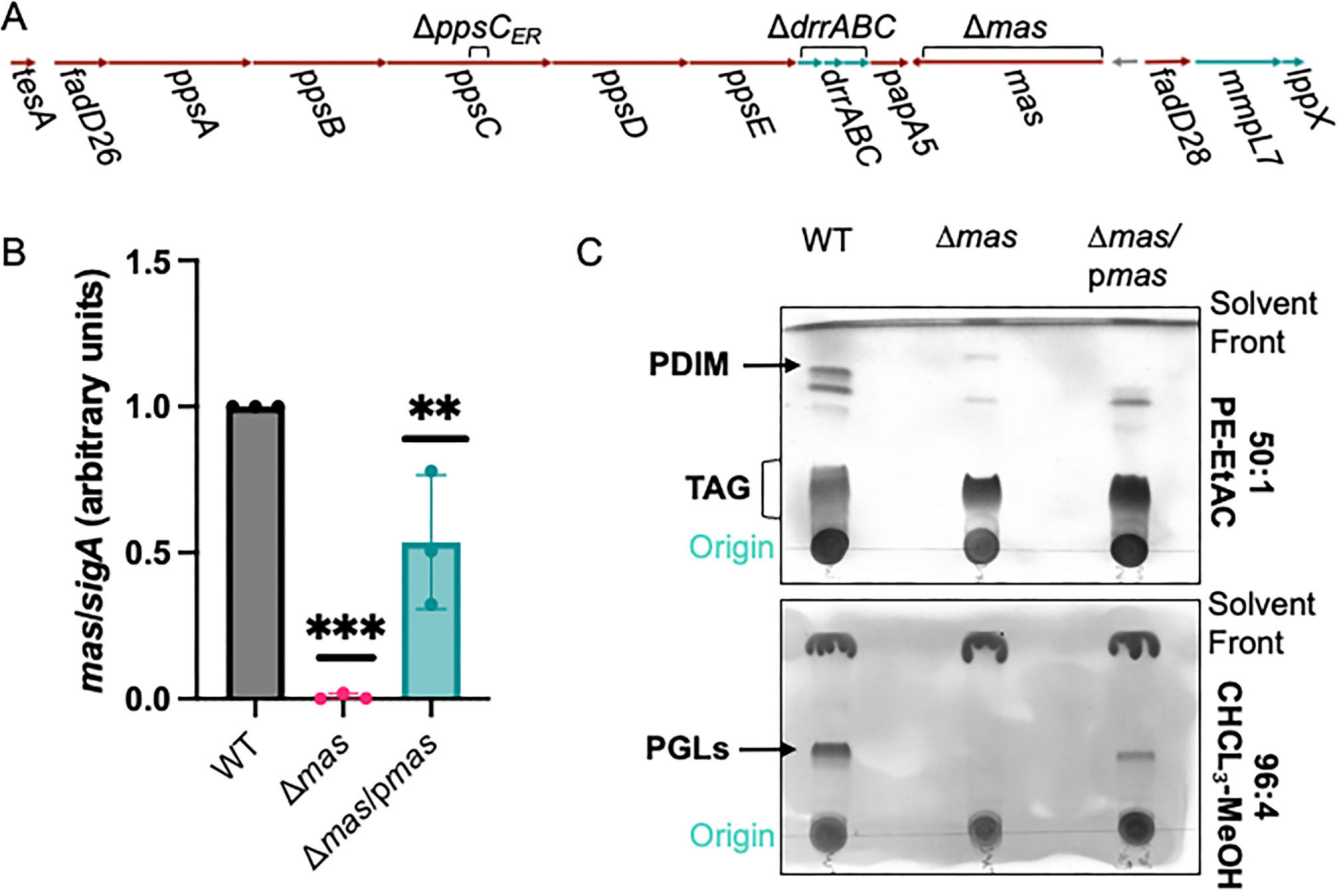
Characterization of PDIM/PGL-deficient *M. marinum* strains. (**A**) Schematic of the genetic loci required for PDIM production (brown) and transport (teal). (**B**) qRT-PCR of the *mas* transcript relative to the *sigA* transcript compared to the WT *M. marinum* strain. Each datapoint is an independent biological replicate and is expressed as an average of three technical replicates. Statistical analysis was performed using an ordinary one-way ANOVA (*P* = 0.0003), followed by Dunnett’s multiple comparison test, ***P = 0.0002; **P = 0.0093. (**C**) TLC of total lipids isolated from the WT, Δ*mas,* and complemented *M. marinum* strains. Nine microliters of total lipids was analyzed. This TLC is representative of at least three independent biological replicates.

The PDIM/PGL biosynthetic pathway is conserved in both *M. tuberculosis* and *M. marinum* (Fig. 1A). We previously constructed *M. marinum* strains with disrupted PDIM production due to deletion of the *mas* gene (49) or the second ER domain of the *ppsC* gene (50). We generated an *M. marinum* strain with an unmarked deletion of the *drrABC* genes. PpsA-E/FadD26 synthesize phthiocerol; loss of the PpsCER domain ablates activity and abrogates PDIM production (6, 50–52). Mas/FadD28 synthesize mycocerosic acid; deletion of *mas* halts PDIM production (2, 12). The two PDIM components are joined by PapA5 and transported by DrrABC, MmpL7, and LppX (2, 14–16). Deletion of *drrABC* abolishes PDIM production and transport (2). Complementation strains were generated by introducing integrating plasmids with the *ppsC_ER_* domain, the *drrABC* operon, or the *mas* gene behind constitutive promoters into each deletion strain. We confirmed the resulting strains using PCR (Fig. S1) followed by targeted DNA sequencing, and qRT-PCR to measure the transcription in the deletion and complementation strains (Fig. 1B; Fig. S2A and B). Deletion of the *mas* or *drrABC* genes abolished the expression relative to the wild-type (WT) and complementation strains. Deletion of the *ppsC_ER_* domain did not impact *ppsC* expression, as expected (50).

We validated PDIM and PGL loss in each deletion strain (Fig. 1C; Fig. S2C) by isolating total soluble lipids and visualizing PDIM and PGL using thin layer chromatography (TLC). All three *M. marinum* deletion strains exhibited a lack of PDIM and PGL (Fig. 1C; Fig. S2C). Reintroducing the *mas* gene restored PDIM in the Δ*mas* strain (Fig. 1C). PDIM and PGL were not complemented by restoring the gene expression in the Δ*ppsC_ER_* and Δ*drrABC* strains (Fig. S2C).

### The loss of PDIM/PGL has a general effect on mycobacterial protein secretion

Past studies linked ESX-1 secretion and PDIM/PGL, proposing that PDIM is necessary for ESX-1 protein secretion (46). However, there have been no studies aimed at understanding how the loss of PDIM/PGL impacts protein secretion more broadly. To clarify the role of PDIM/PGL in mycobacterial protein secretion, we isolated secreted and cell-associated protein fractions and quantified the protein levels in the mutant and complemented strains compared to the WT *M. marinum* strain using Label-Free quantitative (LFQ) proteomics (Data set S1). We measured ESX-1-dependent protein secretion as a control to determine if the impact of PDIM/PGL was limited to ESX-1 secretion.

**Fig 2:**
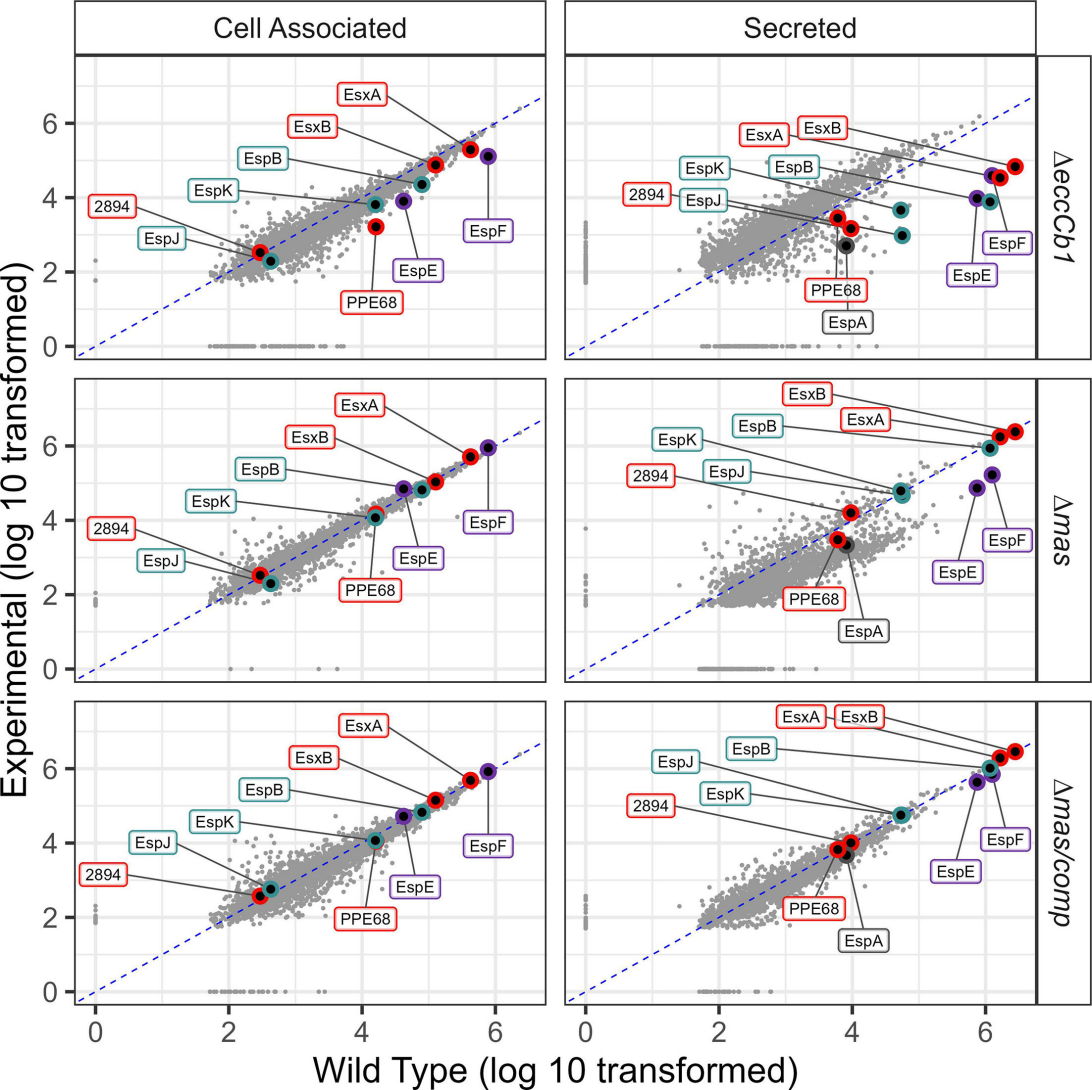
Loss of PDIM/PGL results in widespread changes to protein secretion from *M. marinum*. Label-free quantitative (LFQ) proteomics was used to identify changes in the abundance of proteins in WT, Δ*eccCb_1_*, Δ*mas*, and Δ*mas*/p*mas* strains in both cell-associated (left) and secreted (right) proteomes. Scatter plots of the non-normalized protein peak area, comparing the log_10_ of the WT protein levels (*x*-axis) to the log_10_ of each experimental strain (*y*-axis). Proteins below the dotted line are at lower levels in the experimental strains compared to the WT strain. Values are the average of three technical replicates from three biological replicates. The ESX-1 substrates in Fig. 2 are labeled and color-coded to match Fig. 3A (red, Group I; teal, Group II; purple, Group III).

Deletion of the *eccCb_1_* gene reduced ESX-1 substrate production and secretion (Fig. 2, top, labeled). We reasoned that if PDIM was specifically required for ESX-1 secretion, then deletion of the *mas, ppsC_ER,_* or *drrABC* genes would abolish ESX-1 secretion, similar to the Δ*eccCb_1_* strain. Although deletion of the *mas* gene (or deletion of the *drrABC* genes or the ER domain from the *ppsC* gene) did not cause widespread changes to protein levels (left), it resulted in a significant decrease in the levels of most secreted proteins (proteins below the dotted line in Fig. 2 and in Fig. S3 and S4). Restoring *mas* expression in the Δ*mas* strain complemented the secretion defect (Fig. 2, bottom). These data support that PDIM is important for overall protein secretion from *M. marinum* during laboratory growth.

### The loss of PDIM/PGL specifically impacts the secretion of a subset of ESX-1 substrates

**Fig 3:**
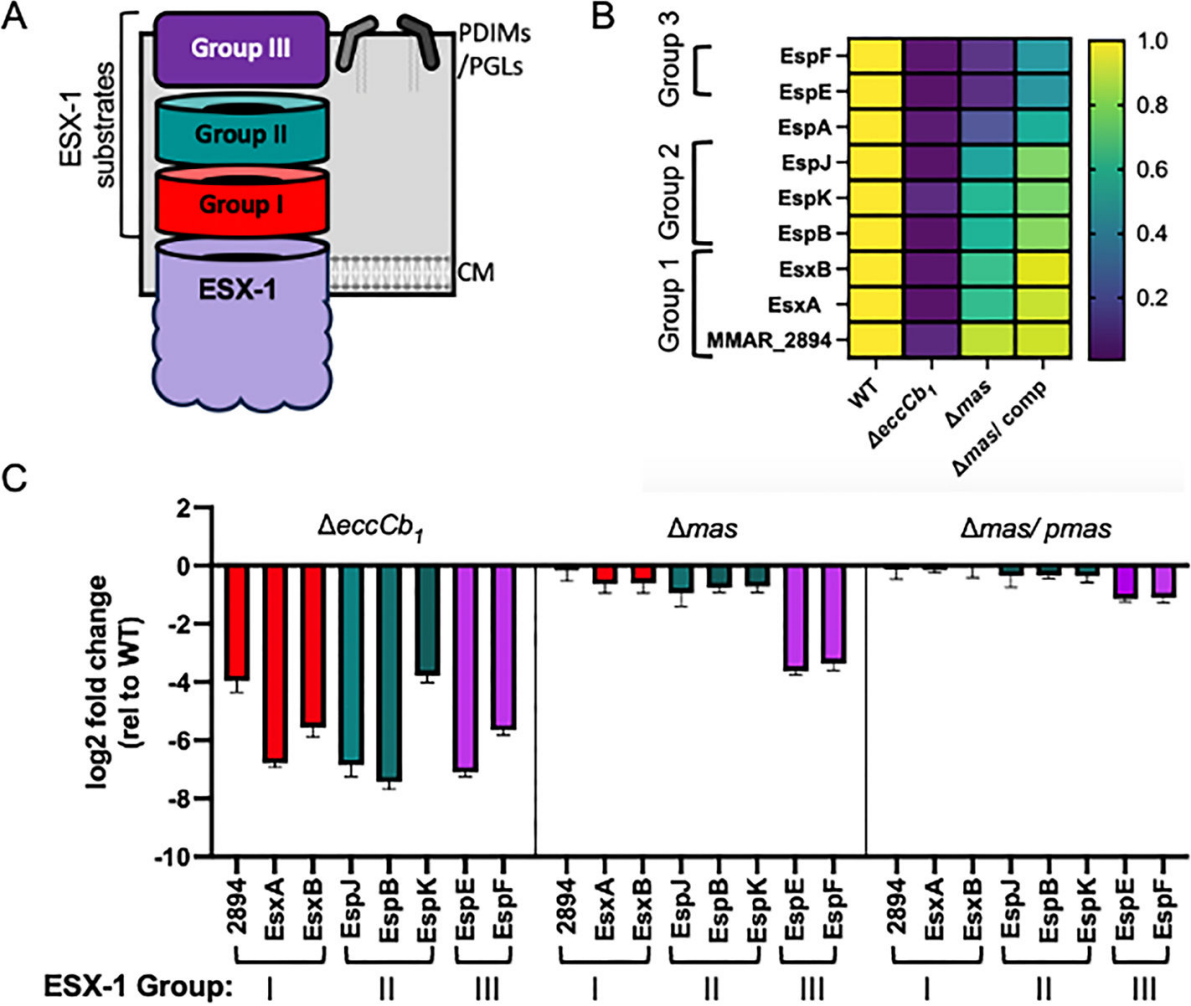
The loss of PDIM/PGL impacts ESX-1 secretion differentially. (**A**) Schematic of the ESX-1 secretion hierarchy. ESX-1 represents the ESX-1 membrane components. Group 1: EsxB, EsxA, PPE68, and MMAR_2894; Group 2: EspB, EspK, and EspJ; Group 3: EspE and EspF, from. (**B**) Heatmap of the ESX-1 substrate levels in the secreted protein fractions normalized to the WT strains (fold change, data in Data Set S1D, from Data Set S1B). (**C**) Log_2_ fold change of ESX-1 substrate levels in the secreted protein fractions compared to the WT strain. Data in Data Set 1C.

We previously demonstrated that ESX-1 substrates are hierarchically secreted from *M. marinum*. We categorized them into at least four groups of ESX-1 substrates (Fig. 3A). Group I substrates include EsxA, EsxB, PPE68, and MMAR_2894, which are essential for the secretion of the other ESX-1 substrates (53, 54). Group II substrates include EspB, EspJ, and EspK (53, 55). These substrates are not required for the secretion of Group I substrates but are essential for the secretion of ESX-1 Group II substrates. ESX-1 Group III substrates include EspE and EspF, and these substrates are dispensable for the secretion of the other classes of ESX-1 substrates. Importantly, deletion of the Group III substrates is sufficient to abrogate the hemolytic activity of *M. marinum* (56).

Figure 3B and C indicates that the loss of PDIM specifically impacts the secretion of ESX-1 Group III substrates. In the Δ*eccCb_1_* strain, ESX-1 substrate levels were significantly reduced (Fig. S5; Data set S1) due to feedback control of substrate gene transcription in the absence of the ESX-1 system (57). However, in the Δ*mas* strain, the substrate levels were not significantly reduced (Fig. S5), indicating no feedback control of substrate gene transcription due to the loss of the PDIM/PGL lipids. While ESX-1 substrate secretion was significantly reduced from the Δ*eccCb_1_* strain because EccCb_1_ is directly required for substrate secretion (58–60), the levels of ESX-1 substrates secreted from the Δ*mas* strain differed, aligning with previously established substrate groups (Fig. 3B and C; Data Set S1). The Group III substrates (EspE and EspF) were the most affected by PDIM/PGL loss. The reduced secretion of Group III substrates was complemented by the expression of *mas* in the Δ*mas* strain (Fig. 3B and C). Together, these data support that the loss of PDIM/ PGL differentially impacts the secretion of the known ESX-1 substrates.

### The absence of PDIM reduces ESX-1 function

**Fig 4:**
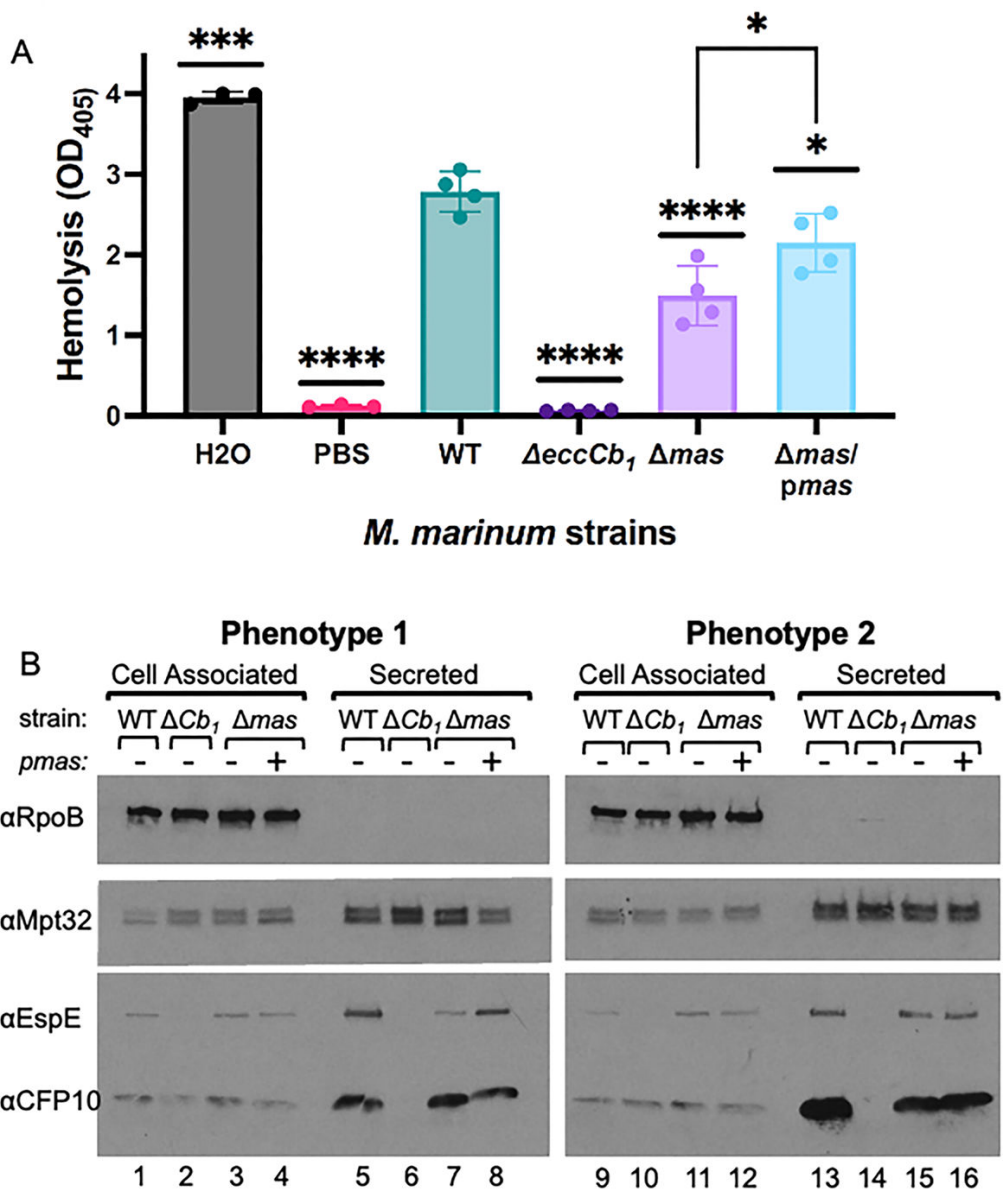
The loss of PDIM/PGL impacts hemolysis and the secretion of the EspE substrate. (**A**) Hemolytic activity of *M. marinum.* Each data point is the mean of three technical replicates. Significance was determined using a one-way ANOVA, *P* < 0.0001, followed by Tukey’s multiple comparisons test ***P = 0.0002, ****P < 0.0001, *P = 0.0259 (Δ*mas*/*pmas* vs WT), and *P = 0.0186 (Δ*mas*/*pmas* vs Δ*mas*). (**B**) Western blot analysis of *M. marinum* cell-associated and secreted protein fractions. Ten micrograms of the protein was loaded in each lane. RpoB is a control for lysis, and MPT32, a protein secreted by the Sec system, is a loading control. The two blots shown were representative of at least four biological replicates. Phenotype 1 refers to the reduced EspE secretion, and phenotype 2 refers to wild-type EspE secretion levels.

Prior research demonstrated that PDIM-deficient *M. marinum* strains exhibited lower ESX-1-dependent hemolytic activity (61). Secretion of EspE and EspF was most impacted by PDIM/PGL loss. Deleting *espE* or *espF* from *M. marinum* abrogated hemolytic activity and resulted in attenuation during macrophage infection (56). Because deleting *espE* or *espF* did not significantly affect the secretion of the additional known ESX-1 substrates, EspE and EspF are at the top of the hierarchy. In Fig. 4A, WT *M. marinum* lysed sheep red blood cells (sRBCs) in a contact-dependent, ESX-1-dependent manner. The Δ*eccCb_1_* strain, which lacks ESX-1 secretion, had significantly less hemolytic activity than the WT strain (*P* < 0.0001) (54, 56). The Δ*mas* strain exhibited intermediate hemolytic activity, between the WT (*P* < 0.0001) and Δ*eccCb_1_* strains (*P* < 0.0001), similar to a prior report (61). Reintroducing the *mas* gene in the Δ*mas* strain significantly increased hemolysis (*P* < 0.0001), but not to the levels found in WT strains (WT vs Δ*mas*/p*mas, P* < 0.0001*)*. Notably, deletion of either the *drrABC* genes or the ER domain of *ppsC* resulted in intermediate hemolytic activity, not significantly different from the Δ*mas* strain (Fig. S2D). Importantly, the reduced hemolytic activity of the PDIM/PGL-deficient strains was still dependent on the ESX-1 system. Deletion of the *eccCb_1_* gene in the Δ*mas* or Δ*drrABC* strains abrogated hemolytic activity (Fig. S6).

We next used Western blot analysis to examine ESX-1 substrate secretion to supplement our proteomics data. EsxB (CFP-10) was produced (Fig. 4B, lane 1) and secreted (lane 5) from the WT strain. EsxB production was reduced (lane 2), and it was not secreted (lane 6) from the Δ*eccCb_1_* strain, as previously reported (50). EsxB production (lanes 3 and 4) and secretion (lanes 7 and 8) were not noticeably impacted in the Δ*mas* and Δ*mas* complemented strains. EspE was produced (lane 1) and secreted (lane 5) from the WT strain. EspE levels were reduced in (lane 2), and EspE was not secreted from the Δ*eccCb_1_* strain (lane 6). Although EspE was made to WT levels in the Δ*mas* strain (lane 3), its secretion was reduced (lane 7). EspE secretion was restored in the Δ*mas*/p*mas* strain (lane 8). These data are consistent with the LFQ proteomics data in Fig. 2 and 3. Interestingly, as we replicated the Western blot analysis, we sometimes observed no visible secretion phenotype, as shown in Fig. 4, right (lanes 7 and 15). The LFQ analysis did not reveal WT levels of EspE and EspF in any of the replicates we tested. From these data, we conclude that Western blot analysis, which is not quantitative, may lack the sensitivity to detect intermediate but biologically relevant changes in ESX-1 protein secretion, as supported by our prior work (53).

## DISCUSSION

This study aimed to understand the impact of the outer lipids PDIM and PGL on ESX-1 secretion. We measured a broad and significant reduction in *M. marinum* protein secretion in the absence of PDIM and PGLs. Interestingly, the loss of PDIM and PGLs differentially impacted ESX-1 protein secretion, particularly affecting the secretion of the Group III substrates, EspE and EspF. Our findings support that the ESX-1-associated phenotypes in strains lacking PDIM/PGL are due to the reduced secretion of specific ESX-1 substrates.

Our unbiased approach to measuring protein secretion revealed that losing PDIM/PGL broadly impacts protein secretion. Contrary to expectations based on the role of PDIM in maintaining envelope impermeability, making the cell envelope permeable did not increase protein secretion by increasing cellular lysis. Instead, overall protein secretion in the culture supernatants decreased, despite evidence of cellular lysis. We do not understand how removing PDIM/PGLs from the MOM results in reduced secretion. The MOM is an asymmetric membrane, and it has been established that the asymmetry of the *E. coli* outer membrane has direct implications on the folding of proteins localized to the OM (62). It is possible that the absence of PDIM or PGL from the MOM impacts protein folding, which could directly or indirectly impact protein secretion.

More specifically, our study has implications that may help clarify the relationship between PDIM/PDL and ESX-1 secretion and mycobacterial pathogenesis. At least three publications previously linked PDIM to ESX-1 secretion or function (46, 47, 61). Barczak et al. concluded PDIM is required for ESX-1 substrate secretion in *M. tuberculosis*. Deletion of *drrC* abrogated EsxA (ESAT-6) and EsxB (CFP-10) secretion and of the ESX-5 substrate, EspN, during laboratory growth. Other PDIM-deficient strains in this study had WT levels of EsxB secretion. The loss of ESX-1 secretion led to reduced IFN induction during macrophage infection with the Δ*drrC* strain, as compared to the WT and complemented strains. IFN induction requires ESX-1-induced lysis of the phagosome membrane (35, 44). It was concluded that the PDIM lipid at the cell surface was important for ESX-mediated protein secretion (46).

In the study by Barczak et al., an intermediate secretion signal may have been missed because the amount of protein loaded in that study was based on the culture density, as measured by OD_600_ (46). We normalized the protein levels based on protein concentration, as measured by the BCA assay because the OD_600_ of *M. marinum* grown in Sauton’s media with low Tween-80 is an unreliable measure in our hands. Using this approach, we did not observe significant differences in the secretion of the Group 1 substrates, including EsxB, by Western blot analysis or using LFQ proteomics. Importantly, Barczak et al. observed variability in EsxB secretion by different PDIM-deficient strains (Δ*drrC* vs Δ*drrA*). Our study revealed that reduced EspE secretion was variable when detected by Western blot analysis, but not by proteomics. We think the inconsistency in measuring these secretory phenotypes by Western blot likely stems from their intermediate nature.

Quigley et al. connected PDIM transport to ESX-1 dependent events during macrophage infection, including phagosome lysis and the resulting macrophage cell death (47). They showed significantly reduced phagosomal lysis of an Δ*mmpL7 M. tuberculosis* strain compared to the WT and complemented strains. Aligned with our studies, Quigley et al. loaded 10 µg of the protein and found no defect in EsxA secretion by Western blot analysis.

Osman et al. demonstrated that PDIM transport was important for phagosomal lysis, finding reduced phagosomal lysis of an Δ*mmpL7 M. marinum* strain during macrophage infection (61). Although the Δ*mmpL7* strain exhibited intermediate hemolytic activity between the ΔRD1 and WT strains, their data did not meet statistical significance. They suggested that PDIM enhanced the ESX-1 lytic activity but proposed that PDIM may contribute differently to hemolysis and phagosomal lysis. Our results show that *M. marinum* strains lacking PDIM/PGL production (Δ*mas* or Δ*ppsC*) or transport (Δ*drrABC*) have significantly reduced hemolysis compared to the WT strain and significantly higher hemolysis compared to the Δ*eccCb_1_ M. marinum* strain. We assessed hemolysis 90 minutes after initiating contact between *M. marinum* and the sRBCs, while Osman et al. measured hemolysis after 2 hours (61). We previously found intermediate hemolysis phenotypes are most apparent at earlier timepoints (57, 63). We suspect that the intermediate hemolytic activity of the Δ*mmpL7* strain would have reached significance relative to the WT strain at earlier timepoints.

An underlying commonality in all of these studies is that in strains lacking PDIM or PGL, ESX-1 activity is reduced, as reflected in contact-dependent hemolytic activity or phagosomal lysis during *M. marinum* or *M. tuberculosis* infection. Notably, at the time of writing of these manuscripts, the secretory relationship between ESX-1 substrates was unclear. However, we now know that EspE/EspF secretion is essential for *M. marinum* hemolysis and for pathogenesis in macrophages (56). We have likewise defined a hierarchical secretory relationship between the ESX-1 substrates (). Here, we showed that the loss of PDIM specifically reduced EspE/EspF (Class III) secretion from *M. marinum*. Because Class III substrates are not required for the secretion of the earlier substrate classes (Class I and Class II) and deletion of either *espE* or *espF* gene abrogates *M. marinum* hemolysis, the secretion of these proteins likely specifically correlates with hemolytic activity (56). We further showed that the hemolytic activity in the absence of PDIM/PGL is still dependent on the ESX-1 system. Therefore, we hypothesize that reduced EspE/EspF secretion caused the reduced hemolytic and phagolytic activity observed in the absence of PDIM in *M. marinum*. It is tempting to speculate that the secretion of proteins, for example, the Group III ESX-1 substrates, that colocalize with PDIM and PGL in the mycobacterial envelope is impacted by the loss of these lipids in *M. marinum*. More recent studies have suggested that PDIM from both *M. marinum* and *M. tuberculosis* spreads into host membranes within macrophages and in animal hosts (7, 8, 64, 65). It is possible that PDIM could spread the Group III ESX-1 substrates or other secreted proteins into host membranes, enhancing ESX-1-contact-dependent lysis. Note, however, that Augenstriech et al. investigated the connection between PDIM and EsxA in the lytic activity of *M. tuberculosis.* In their work, they found that PDIM deficiency had a larger impact on *M. tuberculosis* hemolytic activity than ESX-1 or EsxA, specifically (7). However, it is difficult to compare the hemolytic potential of *M. marinum* and *M. tuberculosis* because the timescale of the assays differs greatly (90 minutes vs 48 hours) and the reporting of hemolysis in *M. tuberculosis* tends to be presented as % WT hemolysis, rather than as OD_405_. These ideas, including the conservation of these findings in *M. tuberculosis,* require further exploration beyond these initial studies.

Taken together, our study has direct implications for mycobacterial pathogenesis, as PDIM- and PGL-deficient *Mycobacterium* are attenuated in cellular infection models (1, 3, 51). Further study is needed to understand if the observed phenotypes due to the loss of mycobacterial outer lipids stem from changes in the secreted proteome.

## MATERIALS AND METHODS

### Growth of bacterial strains

*Mycobacterium marinum* strains were derived from the *Mycobacterium marinum* M strain (ATCC BAA-535). *M. marinum* strains were maintained at 30°C in either Middlebrook 7H9 Broth Base (Sigma Aldrich) supplemented with 0.5% glycerol and 0.1% Tween-80 (Fisher Scientific) or Middlebrook 7H11 agar (Sigma Aldrich) plates supplemented with 0.5% glucose and 0.5% glycerol. Where appropriate, strains were supplemented with 50 µg/mL hygromycin B (Corning, Corning, NY), 20 µg/mL kanamycin (IBI Scientific, Dubuque, IA), and 60 µg/mL X-Gal (RPI, Mount Prospect, IL). *Escherichia coli* DH5α strains were grown in Luria–Bertani Broth (VWR) at 37°C and supplemented with 200 µg/mL hygromycin and 50 µg/mL kanamycin, where appropriate.

### Construction of *M. marinum* strains

The Δ*drrABC* strain was generated using allelic exchange using the p2NIL vector (Addgene plasmid number 20188; a gift from Tanya Parish [66]) and the pGOAL19 vector (Addgene plasmid number 20190; a gift from Tanya Parish [66]), as previously published (54, 56, 57, 63, 67). Primers were purchased from Integrated DNA Technologies (IDT, Coralville, IA) and are listed in Table S2. The complementation plasmids were generated using FastCloning (68) using *M. marinum* M genomic DNA. Plasmids were introduced into *M. marinum* using electroporation, as described previously (54, 56, 57, 63, 67, 69). Strains and plasmids are listed in Table S1. Plasmids and genetic deletions were confirmed by targeted DNA sequencing performed by the Notre Dame Genomics and Bioinformatics Facility.

### Thin-layer chromatography (TLC) of PDIM and PGL lipids

Five-milliliter cultures of *M. marinum* were grown in 7H9 defined broth (Sigma Aldrich) for 2–3 days until turbid, diluted in 50 mL of 7H9 defined broth to an OD_600_ of 0.05, and grown for 72 hours. Mycobacterial cells were harvested via centrifugation (4,000 rpm for 10 minutes). The resulting cell pellets were washed three times with phosphate-buffered saline (PBS). Lipids were extracted using a chloroform (Ricca Chemical Company, Arlington, TX) :methanol (Millipore Sigma) mixture (2:1) overnight in a fume hood. Overnight extraction mixtures were filtered through 6-mm Whatman filter paper (Millipore Sigma) using a porcelain Buchner funnel before separating aqueous and organic phase layers. Extracted lipids were moved to a clean glass vial and dried overnight in a fume hood under a gentle stream of air. Dried mycobacterial lipids were resuspended in a 100-µL mixture of chloroform:methanol (2:1) prior to analysis.

Nine microliters of total mycobacterial lipids was spotted onto aluminum-backed silica TLC plates (Millipore Sigma) and dried. To separate mycobacterial phthiocerol dimycocerosates (PDIM) and triacylglycerols (TAG), spotted lipids were migrated three times sequentially in a running solution of 50:1 petroleum ether:ethyl acetate (Millipore Sigma). The TLC plate was sprayed with phosphomolybdic acid (Millipore Sigma, concentration greater than or equal to 5%, but less than 10% phosphomolybdic acid hydrate in isopropanol) and charred via a heat gun (Westward) to visualize the lipids.

Mycobacterial phenolic glycolipids (PGLs) were visualized using 9 µL of total mycobacterial lipids migrated once in a solution of 96:4 chloroform and methanol. The TLC plate was sprayed with a solution of 1% α-naphthol (TCI Chemicals, Portland, OR) and charred via a heat gun to visualize the lipids.

### Hemolysis assays

Hemolysis assays were performed exactly as described () except that sheep red blood cell and bacteria were incubated at 30**°**C for 1.5 hours, rather than 2 hours.

### Secretion assays

Cell-associated and secreted protein fractions were prepared from *M. marinum* exactly as previously described. Briefly, *M. marinum* bacterial cultures were grown in 7H9 defined media, diluted in 50 mL of Sauton’s media with 0.01% Tween at an OD_600_ of 0.8, and grown for 48 hours before harvesting cellular and secreted fractions by centrifugation. Culture supernatants were filtered through 0.2-µm Nalgene Rapid-Flow Bottle Top Filters with PES membranes (Thermo Scientific) and concentrated using 15 mL, 3 kDa Amicon Ultra Centrifugal Filter Units (Millipore Sigma). Mycobacterial cell pellets were resuspended in 0.5 mL of PBS, lysed via bead-beating with a BioSpec Mini-Beadbeater-24, and clarified by centrifugation. Protein concentrations of the resulting fractions were measured using the Pierce Micro BCA Protein Assay Kit (Thermo Scientific).

### Western blot analysis

Ten micrograms of each protein sample was loaded into 4–20% TGX Gradient Gels (Bio-Rad) for analysis. Antibodies were diluted in 5% non-fat dry milk (RPI) in PBS with 0.1% Tween-20 (VWR). Rpoβ (anti-RNA polymerase beta mouse monoclonal antibody [clone: 8RB13]; VWR) was diluted 1:20,000. The following reagents were obtained through BEI resources, NIAID, NIH: polyclonal anti-*Mycobacterium tuberculosis* CFP-10 (gene Rv3874; antiserum, rabbit; NR-13801) and polyclonal anti-*Mycobacterium tuberculosis* Mpt-32 (gene Rv1860; antiserum, rabbit; NR-13807). CFP-10 was used at a 1:5,000 dilution. Mpt-32 was used at a 1:30,000 dilution. The EspE antibody (1:5,000 dilution) was a custom rabbit polyclonal antibody against the CGQQATLVSDKKEDD peptide (Genscript).

### Proteomics/LC-MS

LC-MS pure reagents (water, ethanol, acetonitrile, and methanol) were purchased from J. T. Baker (Radnor, PA). Iodoacetamide (IAA) was purchased from MP Biomedicals (Solon. OH). All other reagents were obtained from Millipore-Sigma (St. Louis, MO), unless specified. S-Trap mini devices were obtained from Protifi (Huntington, NY). Trypsin Gold was purchased from Promega (Madison, WI). Hydrophilic–lipophilic balance (HLB) solid-phase extraction (SPE) cartridges (1 cc/10 mg) from Waters (Milford, MA) were used to desalt peptide samples prior to analysis on a timsTOF Pro from Bruker Scientific LLC (Billerica, MA). Protein and peptide identification including label-free quantitation was performed using the PEAKS Online X search engine from Bioinformatics Solutions Inc. (Waterloo, ON) (build 1.4.2020-10-21_171258).

Cell-associated and secreted cell lysate samples were prepared for LC-MS analysis, as described (49, 70). Fifty micrograms of each sample was prepared in 100 mM triethylammonium bicarbonate (TEAB), 5% sodium dodecyl sulfate (SDS), and 100 mM tris(2-carboxyethyl)phosphine (TCEP). Samples were heated for 10 minutes at 95°C, cooled, and then IAA was added to 100 mM IAA for 30 minutes in dark.

Samples were acidified with *o-*phosphoric acid to a final concentration of 1.2% in 50 µL and then flocculated with 350 µL binding buffer containing 90% methanol and 10% 1M TEAB. Samples were passed through S-Trap Micro filters and followed by washing two times in 80 µL of binding buffer and 80 µL of 1:1 methanol/chloroform solution. A new collection tube was added, and 1 µg of trypsin in 80 µL 100 mM TEAB was added. Samples were wrapped in parafilm to prevent evaporation and incubated at 37°C for 12 hours. Digested peptides were spun through the filter, followed by two 80 µL elutions with 0.1% formic acid in water and one 80 µL elution with 0.1% formic acid in 50% acetonitrile. Eluted peptides were vacuum-concentrated for 20 minutes (to remove acetonitrile) and then desalted using 1 cc/10 mg HLB SPE cartridges following the manufacturer’s specifications and then dried by a vacuum concentrator prior to analysis.

Desalted peptides were resuspended in 0.1% formic acid and water to a concentration of 1 mg/mL. One hundred nanograms of each sample was injected in triplicate onto an Evosep One and timsTOF Pro LC-MS system. Each sample was prepared in biological triplicate and technical triplicate. 15spd (Sample-per-day) methods were used on a 150 µm x 150 mm PepSep column with the C_18_ ReproSil AQ stationary phase at 1.9 mm particle size, 120 Å pore size (manufacturer’s protocol). Samples were loaded using Evotips following the manufacturer’s specifications. Nano-ESI was used with a spray voltage of 1,700 V. MS was set to the parallel accumulation, serial fragmentation data-dependent mode (PASEF-DDA) with a mass range of 100–1,700 *m*/*z*, ion mobility range of 0.6–1.6 v*s/cm^2^, and ramp and accumulation times of 100 ms. Each precursor consisted of 10 PASEF ramps for a cycle time of 1.17 s. Precursors were filtered to contain only charges from 2 to 5. MS/MS collision energy settings were set to ramp from 20 eV at 0.6 ion mobility to 70 eV at 1.6 ion mobility. Instrument tune parameters were set to default for proteomic studies with the following differences: quadrupole low mass set to 20 *m*/*z* and focus pre-TOF pre-pulse storage set to 5 ms.

Protein and peptide identification and label-free quantitation were performed using the PEAKS Online X search engine. The search database used was the *M. marinum* proteome from Mycobrowser (v4 release). Search settings were set to manufacturer defaults unless specified. Three missed cleavages were allowed, at a semi-specific search. Fixed modification included carbamidomethylation of cysteine, while variable modifications included protein N-terminal acetylation, deamidation of asparagine and glutamine and pyroglutamic acid from glutamine and glutamic acid, and oxidation of methionine. All peptide-spectrum matches were filtered to a 1% false discovery rate. Cell-associated samples were normalized by total ion current (TIC).

LFQ search results were trimmed as follows. Technical replicate values for each sample were averaged if at least two of the three replicates were nonzero values. The same averaging logic was applied to biological replicates. Only nonzero values were averaged. All mutant samples were ratioed to the corresponding WT values to generate fold change ratios.

### Data analysis

Peak protein outputs for each injection were analyzed and visualized in R (4.0.3) using the following packages: dplyr (1.1.3), tidyr (1.3.0), ggplot2 (3.4.4), stringr (1.5.0), and ggrepel (0.9.4) using the protein area measurement for abundance. For each comparison (WT vs deletion or complement strain), proteins were included if there were three identifications among all biological and technical replicates (*n* = 6 for ∆*eccCb1, n* = 9 for all others) for WT and/or experimental. All technical and biological replicates were used to calculate the fold change (experimental/wild-type) and significance by pairwise *t*-tests. Significance values were corrected using the Benjamini–Hochberg (B-H) method for multiple hypothesis testing using a false discovery rate of α = 0.05. For proteins where one condition contained one or two measurements (compromised values), a fold change was calculated but significance could not be determined, so these fold change values were placed at *y* = 0 on volcano plots. Proteins with infinite and negative infinite fold changes (observed in one condition and not another) were placed at the minimum and maximum *x*-axis values on volcano plots with random y-value intensity. On XY intensity plots, these infinite fold changes were placed at 0 on their respective axis. On volcano plots, proteins with log_2_ fold changes of greater than 1 or less than –1 along with a B-H critical value above the significance cutoff were highlighted. These thresholds are shown with dotted lines. On XY intensity plots, the line *y* = *x* is shown with a dotted blue line. The R code and the csv files can be found at https://github.com/Champion-Lab/PDIM.

### RNA extraction

*M. marinum* strains were grown as described in “Secretion assay.” Fifteen milliliters of Sauton’s culture was pelleted by centrifugation. Bacterial pellets were resuspended in Qiagen RLT buffer (Qiagen, Hilden, Germany), supplemented with 1% β-mercaptoethanol. Lysates were generated by bead beating with the Biospec Mini-BeadBeater-24 and clarified with centrifugation. Total RNA was extracted using the RNeasy Mini Kit (Qiagen), according to the manufacturer’s instructions.

### qRT-PCR

RNA (500 ng) was treated with RQ1 DNase (Promega) according to the manufacturer’s instructions. One microliter of DNase-treated RNA was converted to cDNA using random hexamers (IDT) and SuperScript II Reverse Transcriptase (Invitrogen) according to the manufacturer’s instructions. cDNA was quantified using a NanoDrop 2000 (Thermo Scientific, Waltham, MA); 10 µL qRT-PCR mixtures were prepped with 250 ng of cDNA, SYBR Green PCR Master Mix (Applied Biosystems, Carlsbad, CA) and 1 µM of each oligonucleotide. Reactions were run using Applied Biosystems MicroAmp Fast 96-well plates (0.1 mL) (Life Technologies). Plates were run on the QuantStudio 3 Real-Time PCR system (Thermo Fisher). Cycle conditions were as follows: 50°C for 2 min, 95°C for 10 min, then 40 cycles at 95°C for 15 s and 60°C for 1 min, and then a dissociation step of 95°C for 15 s, 60°C for 1 min, 95°C for 15 s, and 60°C for 15 s.

qRT-PCRs were analyzed using ΔΔCt comparisons. qRT-PCR results were normalized to WT transcript abundance using the following equations:


$$\begin{array}{c} \Delta Ct=Ct(gene\;of\;interest)-Ct(housekeeping\;gene)\\ Then:\\ \Delta \Delta Ct=\Delta Ct(treated\;sample)-\Delta Ct(untreated\;sample)\\ Then:\\ 2^{-\Delta \Delta Ct}=fold\;change \end{array}$$

## Data Availability

RAW files are deposited and available at MassIVE (ftp://MSV000093713@massive.ucsd.edu; MSV000093713) and Proteome Exchange (PXD048051). The R code and the csv files can be found at https://github.com/Champion-Lab/PDIM.

## SUPPLEMENTAL MATERIAL

Data Set S1 msphere.00005-24-s0001.xlsx Proteomics data.

Supplemental Material msphere.00005-24-s0002.pdf Figures S1-S6 and Tables S1 and S2.

ASM does not own the copyrights to Supplemental Material that may be linked to, or accessed through, an article. The authors have granted ASM a non-exclusive, world-wide license to publish the Supplemental Material files. Please contact the corresponding author directly for reuse.
