# Supplementary material for: The loss of the PDIM/PGL virulence lipids causes differential secretion of ESX-1 substrates in Mycobacterium marinum
Source: mSphere. 2024 Apr 25;9(5):e00005-24. doi: 10.1128/msphere.00005-24 (PMC11237470; doi:10.1128/msphere.00005-24)
Supplement: Supplemental Material — Figures S1-S6 and Tables S1 and S2. [file msphere.00005-24-s0002.pdf]

Supplementary Information for: **The loss of the PDIM/PGL virulence lipids causes differential secretion of ESX-1 substrates in *Mycobacterium marinum***

Bradley S. Jones[*1,2], Daniel D. Hu[*3], Simon D. Weaver[3], Kathleen R. Nicholson[1,4], Rachel M. Cronin[1,5], Matthew M. Champion[2, 3], and Patricia A. Champion[1,2#]

[1]Department of Biological Sciences, [2]Eck Institute for Global Health, [3]Department of Chemistry and Biochemistry, University of Notre Dame, Notre Dame, Indiana, 46556

[4]Current Address: Feinberg School of Medicine, Northwestern University, Chicago, Illinois, 60611

[5]Current Address: Laboratory of Respiratory and Special Pathogens, Division of Bacterial, Parasitic, and Allergenic Products, Office of Vaccines Research and Review, Center for Biologics Evaluation and Research, Food and Drug Administration, Silver Spring, MD, USA

Contents:

Figure S1. Confirmation of genetic deletion and complementation strains
Figure S2. Characterization of the Δ*ppsCER* and Δ*drrABC* strains
Figure S3. Volcano Plots of LFQ Proteomics on cell associated and secreted protein fractions
Figure S4. Scatter Plots of LFQ Proteomics on cell associated and secreted protein fractions from the PDIM/PGL deficient strains.
Figure S5: Representation of ESX-1 substrate levels in cell associated fractions
Figure S6: ESX-1 is essential for hemolysis in the absence of PDIM/PGL.
Table S1: Strains and Plasmids used in this study
Table S2: Oligonucleotide primers used in this study

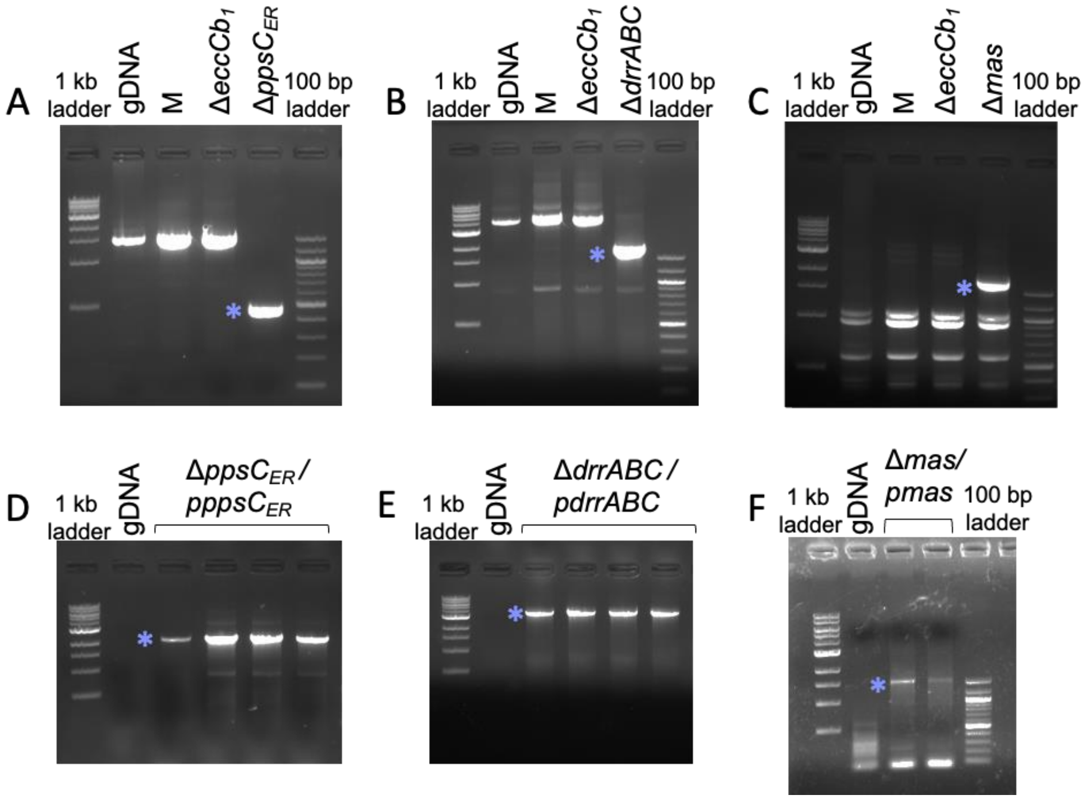

Figure S1. Confirmation of genetic deletion and complementation strains. PCR confirmation of the **A**. Δ*ppsC*$_{ER}$ strain; expected size of WT allele from the M strain, 1,532 bp, Δ, 464 bp, **B.** Δ*drrABC* strain; expected size of the WT allele, 4,507 bp, Δ, 1,833 bp. **C.** Δ*mas* strain; expected size of WT allele, 8,105 bp, Δ, 1,826 bp. PCR for presence of the complementation plasmid in the **D.** Δ*ppsC*$_{ER}$ strain; expected size from plasmid PCR: 2,263 bp. **E.** Δ*drrABC* strain; expected size from plasmid PCR: 3,838 bp. **F.** Δ*mas* strain expected size from plasmid PCR: 1,545 bp.

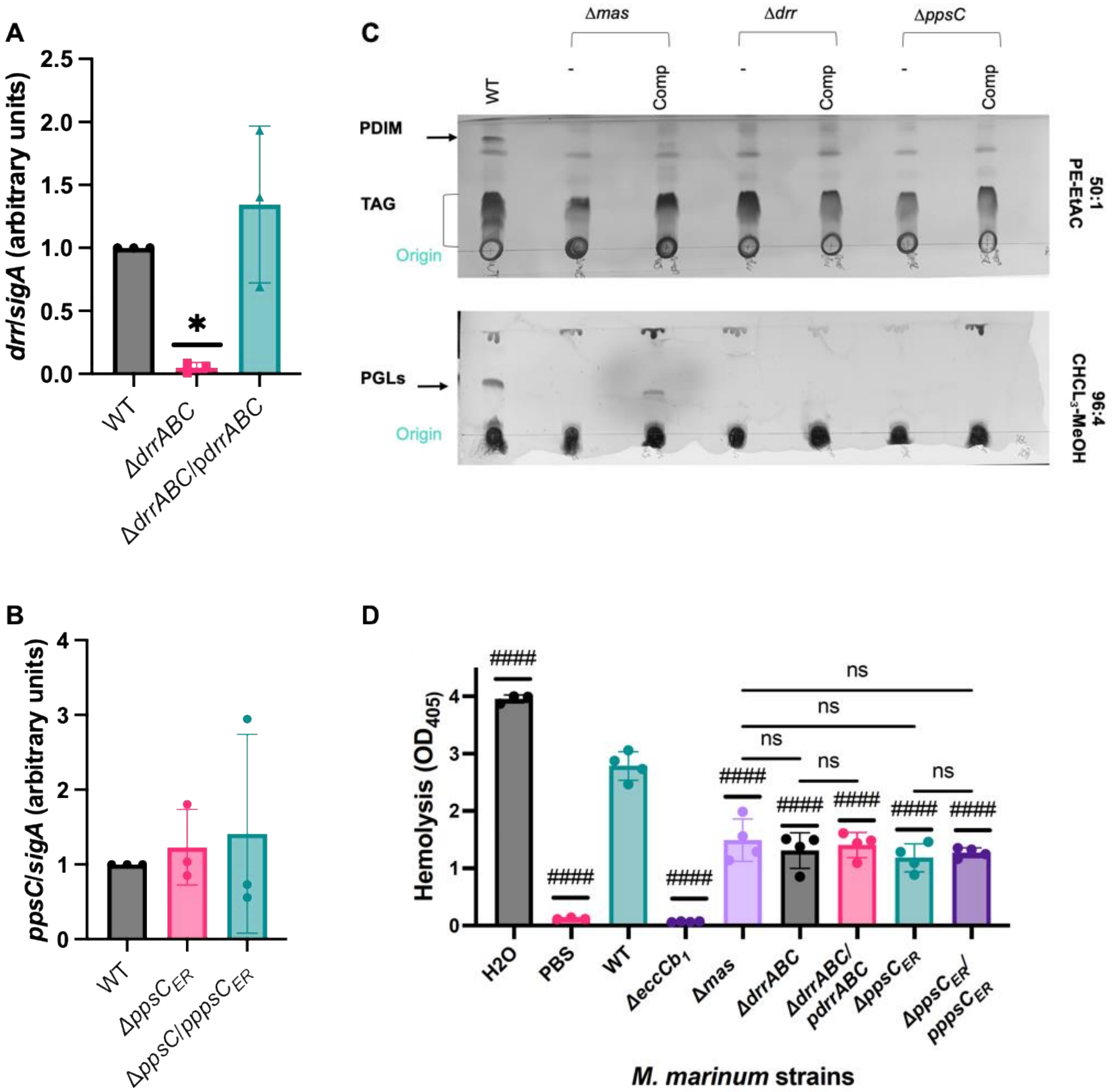

Figure S2. Characterization of the Δ*ppsCER* and Δ*drrABC* strains. qRT-PCR of **A.** *drrA* transcript or **B.** *ppsC* transcript upstream of the ER domain, relative to the *sigA* transcript compared to the WT *M. marinum* strain. Each datapoint is an independent biological replicate, and an average of three technical replicates. Statistical analysis was performed using an ordinary one-way ANOVA (P=.0113 for A, B was not significant), followed by a Dunnett's multiple comparison test, * P=.0316). **C.** TLC of total lipids isolated from the deletion and complementation *M. marinum* strains. 6µl of total lipids was analyzed. This TLC is representative of at least three independent biological replicates. D. Hemolysis of *M. marinum* strains. Each data point represents the mean of three technical replicates from a biological replicate. Statistical analysis was performed using a one-way ordinary ANOVA (P<.0001), followed by a Tukey's multiple comparison test. #### P<.0001 relative to the wild-type strain, ns, not significant.

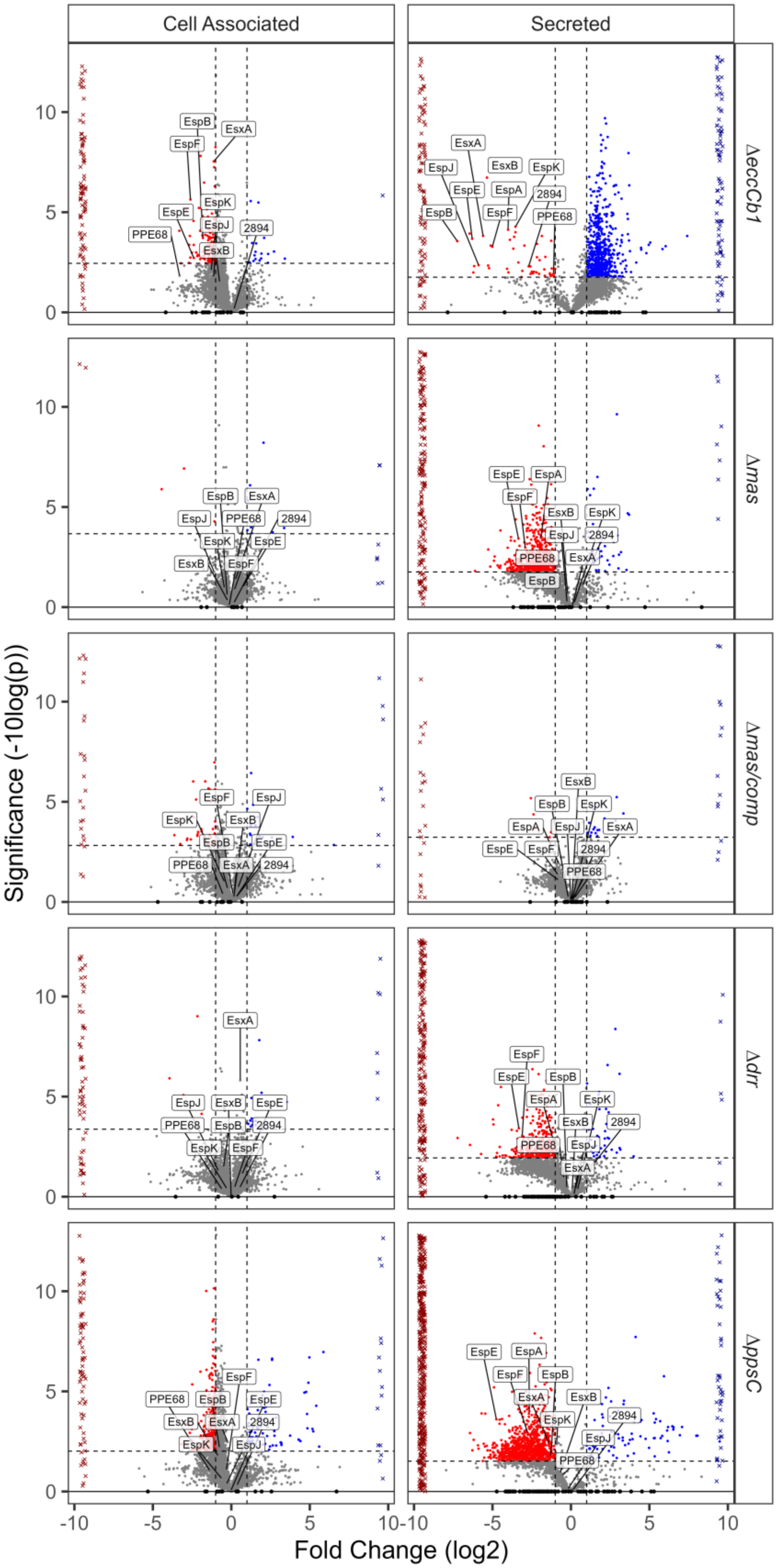

Cell Associated
Secreted
ΔeccCb1
Δmas
Δmas/comp
Δdrr
ΔppsC
Significance (-10log(p))
Fold Change (log2)
EspB
EsxA
EspF
EspE
EspK
EspJ
PPE68
2894
EsxB
EspA

Figure S3. Volcano Plots of LFQ Proteomics on cell associated and secreted protein fractions. Volcano plots of protein levels from cell associated (left) and secreted (right) protein fractions. Values are the average of three technical replicates from three biological replicates. Significance, the -log10 of the p-value calculated from pairwise t-tests, was plotted against the log2 fold change compared to the WT strain. Horizontal dotted line represents the significance cutoff for a Benjamini Hochberg corrected p-value of 0.05. Vertical, black dashed lines signify a log2 foldchange = +/- 1. Red points are significantly decreased in the mutant strains relative to the WT strain. Blue dots are significantly increased in the mutant strains relative to the WT strain. ESX-1 substrates of interest are labeled. Dark red and dark blue "X"s on the left and right of each plot represents infinite fold changes (identified in one strain, but not the other), with y-values randomly assigned. Compromised values (2 or less observed measurements in one strain) are represented as black dots at y=0, where a fold change could not be calculated, but was not high enough to calculate significance. These are the same data represented in the Scatter Plots in Figure 2.

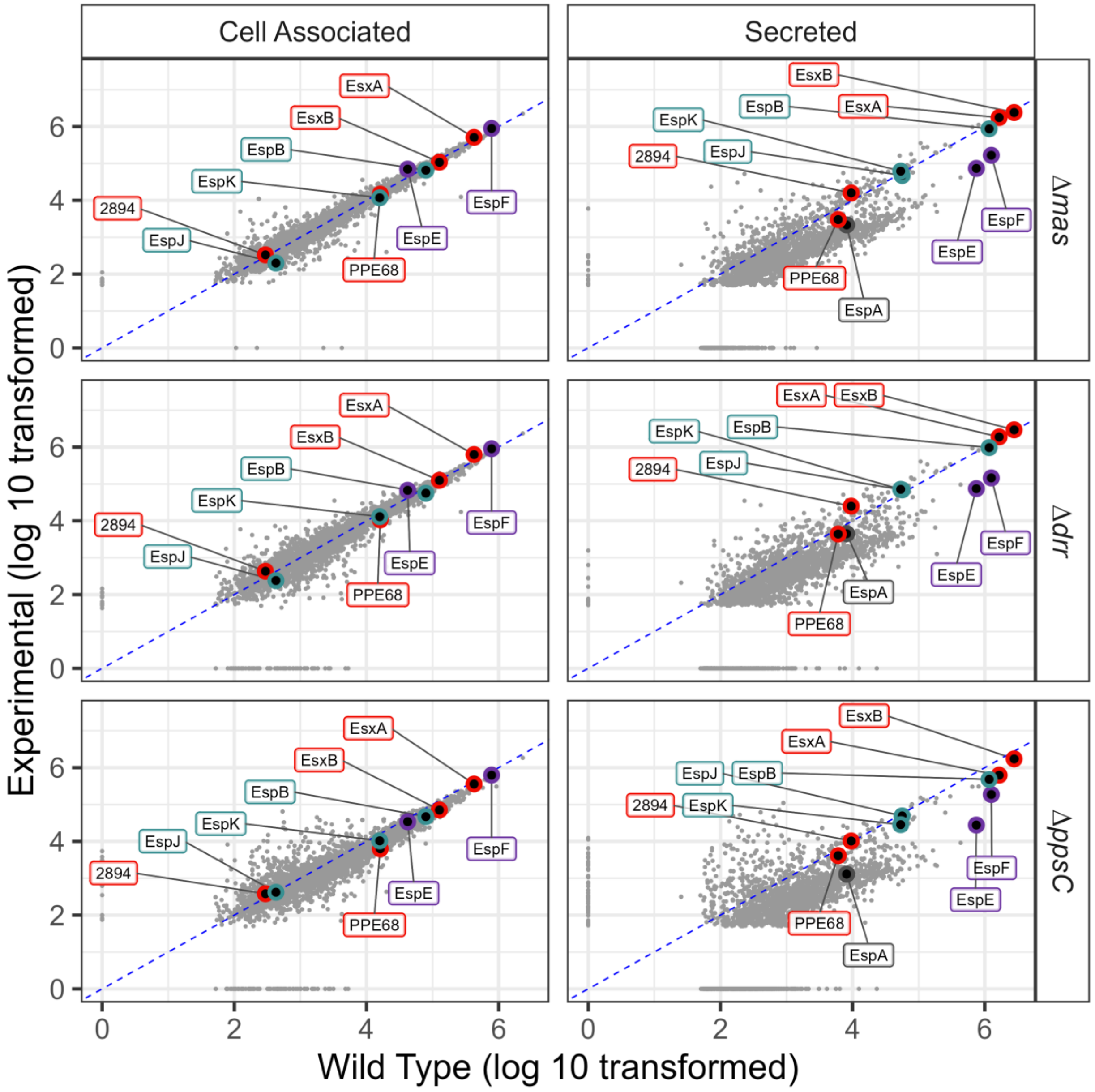

Cell Associated
Secreted
Δmas
Δdrr
ΔppsC
EsxA
EsxB
EspB
EspK
EspJ
EspE
EspF
EspA
PPE68
2894
Experimental (log 10 transformed)
Wild Type (log 10 transformed)
0
2
4
6

Figure S4. Scatter Plots of LFQ Proteomics on cell associated and secreted protein fractions from the PDIM/PGL deficient strains. Label free quantitative proteomics (LFQ) was used to identify changes in the abundance of proteins in Δ*mas*, Δ*drrABC*, and Δ*ppsC*$_{ER}$ strains in both cell-associated (left) and secreted (right) proteomes. Scatter plots are non-normalized protein peak area, comparing the log10 of the WT protein levels (x-axis) to the log10 of each experimental strain (y-axis). Proteins below the dotted line are at lower levels in the experimental strains compared to the WT strain. Values are the average of three technical replicates from three biological replicates. The ESX-1 substrates in Figure 3 are labeled and color coded to match Figure 3 A (red, Group I, teal, Group II, purple, Group III).

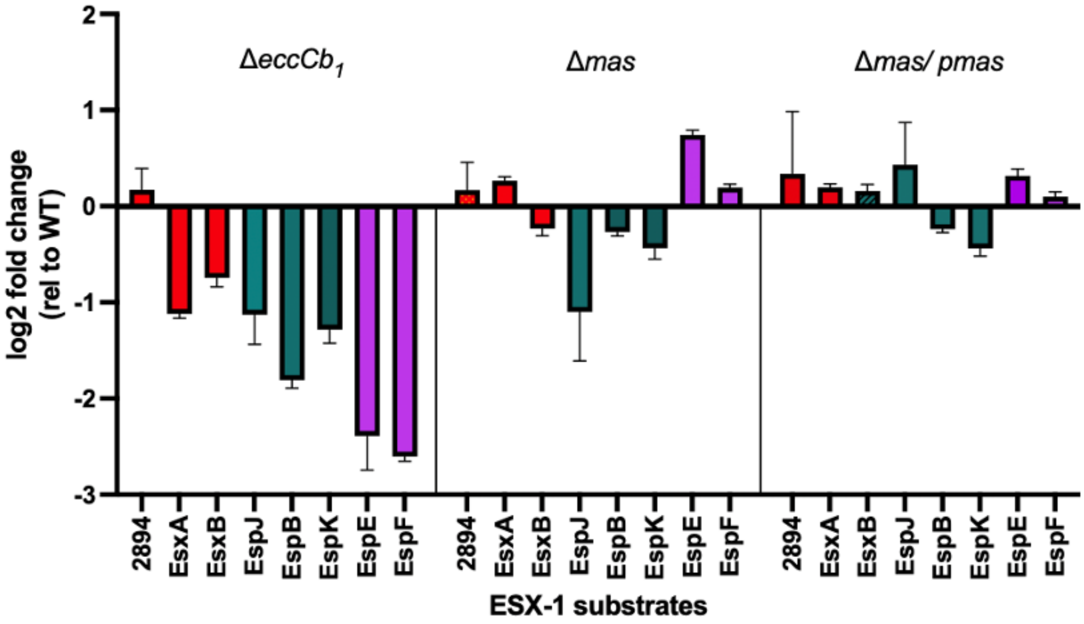

Figure S5. Representation of ESX-1 substrate levels in cell associated fractions. Substrates are separated into their hierarchical group, red = Group I, teal is Group II, and purple is Group III. Reduction in ESX-1 substrate levels in the Δ*eccCb*$_1$ are due to feedback control in the absence of the ESX-1 system. Related to Figure 3.

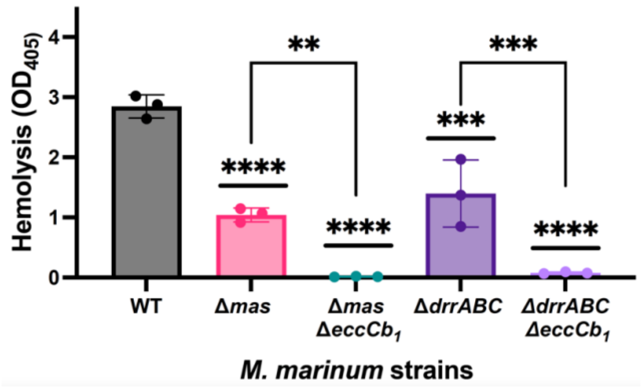

Figure S6. ESX-1 is essential for hemolysis in the absence of PDIM/PGL. Hemolysis of *M. marinum* strains. Each data point represents the mean of three technical replicates from a biological replicate. Statistical analysis was performed using a one-way ordinary ANOVA (P<.0001), followed by a Tukey's multiple comparison test. **** P<.0001, *** P=.0004 (Δ*drrABC* vs WT), P=.001 (Δ*drrABC* vs Δ*drrABC*Δ*eccCb*$_1$), ** P=.0061 (Δ*mas* vs Δ*mas*Δ*eccCb*$_1$).

Table S1. Bacterial strains and plasmids used in this study.

**Strains**	**Genotype**	**Reference**
M	Wild-type *M. marinum,* ATCC: BAA-545	ATCC
Δ*drrABC*	Unmarked deletion of the *drrA* (*MMAR_1771*), *drrB* (*MMAR_1770*), and *drrC* (*MMAR_1769*) genes	This study
Δ*drrABC*Δ*eccCb*$_1$	Unmarked deletions of the *drrA-C* (*MMAR_1771-1769*) and *eccCb*$_1$ (*MMAR_5446)* genes	This study
Δ*eccCb*$_1$	Unmarked deletion of the *eccCb*$_1$ gene (*MMAR_5446)*	(1)
Δ*mas*	Unmarked deletion of the *mas* gene (*MMAR_1767*)	(2)
Δ*mas*Δ*eccCb*$_1$	Unmarked deletions of the *mas* (*MMAR_1767*) and *eccCb*$_1$ (*MMAR_5446)* genes	This study
Δ*ppsC*$_{ER}$	Unmarked deletion of the enoyl reductase domain of the *ppsC* gene (*MMAR_1774*)	(1)
**Plasmids**	**Description**	**Reference**
pMH406 Hyg	pMV306 with *esxBA*$_{MT}$ behind the mycobacterial optimal promoter (mop). L5 origin, integrates at *attB*. Modified from Kan^R to Hyg^R; parental plasmid for the *mas* complementation plasmid	Original reference (3), Hyg^R version (4)
p2NIL	parental suicide vector for allelic exchange, Kan^R, Amp^R	(5), Addgene plasmid #20188
pGOAL19	marker cassette for allelic exchange, amp^R, Hyg^R, *lacZ*+ *sacB*	(5), Addgene plasmid #20190
p*drrABC*	*drrABC* genes behind the *mop* promoter	This study
p*mas*	*mas* gene behind the *mop* promoter	This study
p*ppsC*$_{ER}$	*ppsC*$_{ER}$ domain behind the *mop* promoter	This study

Table S2. Oligonucleotide Primers used in this study.

Primer	Primer Sequence 5'->3'	Purpose	Reference
orb159	ACTGGATTCAGCCGGTGGTG	genotyping Δ*mas*	(2)
orb160	ATCCGGCTTGGCCTGGATTG		
orb161	CATCGGTTGGCGCAAAGCTC	genotyping Δ*drrABC*	This study
orb162	CTCGCTGAATGCCAGCTTCC		
oew268	AACGCTTTGTCCACCGACTG	genotyping Δ*ppsC*$_{ER}$	(1)
oew269	CGAGAAGGTCAGCCACCAATC		
OKN118	gcctgagcggtcccgactagtAGACTCTCCGTTCTTCAC	p*drrABC* complementation plasmid	This study
OKN119	aggagtccagccatATGCGTAACAACGACTTGG		
OKN120	gcctgagcggtcccgactagtGGATCCGCCGTTATGAC	p*mas* complementation plasmid	This study
OKN121	aggagtccagccatGTCGAGCTGAGACAAGG		
OKN132	aggagtccagccatCCGCACCGTGGTAAATCTG	p*ppsC*$_{ER}$ complementation	This study
OKN133	gcctgagcggtcccgactagtACCACGAAACCCAAACC		
OMF630	ACTAGTCGGGACCGCTCAGGCGTCC	Amplification of pMH406H for FastCloning	(4, 6)
OMF057	catatgGCTGGACTCCTGAATTCTGCAGCTG		
oew264	GTGGTGTCACGCTCGTTGGCCTCAAACGCGCATCAG	Generation of Δ*ppsC*$_{ER}$ strain	(1)
oew265	CAAACCGCCCATGCCATTTACCACGGTGCGGCGAGATTCGAG		
oew266	CGCACCGTGGTAAATGGCATGGGCGGTTTGGGTTTC		
oew267	GCAGTCAGGCACCGTTTGCCGTTTCCAGCTCGCTTG		
orb142	TGGTGTCACGCTCGTGCCGCAGGATCTGTATGAAC	Generation of the Δ*drrABC* strain	This study
orb143	TGGTGGATCAATGCGTGTTACGCATCCAATCCTCCTC		
orb144	ACGCATTGATCCACCATGC		
orb145	GCAGTCAGGCACCGTCTTATGGCAGCGAACTAGG		
orb146	TGGTGTCACGCTCGTGCAGTGAAGAACGGAGAGTC	Generation of Δ*mas* strain	(2)
orb147	TCATAACGGCGGATCCGTTG		
orb148	GGATCCGCCGTTATGAGGCAGGTTTTTGCATAAATC		
orb149	GCAGTCAGGCACCGTCGTTGTCGTTCTTGCCATTC		
OKNq50	GAGGATCCGAGGAAGACGAG	*mas* qRT-PCR primers	This study
OKNq51	GATCTGCAGACGCATGTTGT		
OKNq52	ACTTCGTCACCCTCATCGAA	*drrABC* qRT-PCR primers	This study
OKNq53	GATCAACGGAACGATGCTGT		
OKNq56	CTCGACTCGCTGAGTAAGGT	*ppsC* qRT-PCR primers	This study
OKNq57	CTTCATGAATCCGGCGATCC		

Supplementary References

1. E. A. Williams *et al.*, A Nonsense Mutation in Mycobacterium marinum That Is Suppressible by a Novel Mechanism. *Infect Immun* **85** (2017).
2. O. A. Collars *et al.*, An N-acetyltransferase required for ESAT-6 N-terminal acetylation and virulence in Mycobacterium marinum. *mBio* **14**, e0098723 (2023).
3. K. M. Guinn *et al.*, Individual RD1-region genes are required for export of ESAT-6/CFP-10 and for virulence of Mycobacterium tuberculosis. *Mol Microbiol* **51**, 359-370 (2004).
4. K. G. Sanchez *et al.*, EspM Is a Conserved Transcription Factor That Regulates Gene Expression in Response to the ESX-1 System. *mBio* **11** (2020).
5. T. Parish, N. G. Stoker, Use of a flexible cassette method to generate a double unmarked Mycobacterium tuberculosis tlyA plcABC mutant by gene replacement. *Microbiology* **146 (Pt 8)**, 1969-1975 (2000).
6. R. E. Bosserman, K. R. Nicholson, M. M. Champion, P. A. Champion, A New ESX-1 Substrate in Mycobacterium marinum That Is Required for Hemolysis but Not Host Cell Lysis. *J Bacteriol* **201** (2019).
